# Economic and Disease Burden of Dengue in Mexico

**DOI:** 10.1371/journal.pntd.0003547

**Published:** 2015-03-18

**Authors:** Eduardo A. Undurraga, Miguel Betancourt-Cravioto, José Ramos-Castañeda, Ruth Martínez-Vega, Jorge Méndez-Galván, Duane J. Gubler, María G. Guzmán, Scott B. Halstead, Eva Harris, Pablo Kuri-Morales, Roberto Tapia-Conyer, Donald S. Shepard

**Affiliations:** 1 Schneider Institutes for Health Policy, Heller School, Brandeis University, Waltham, Massachusetts, United States of America; 2 Carlos Slim Health Institute, Mexico City, Mexico; 3 Instituto Nacional de Salud Pública, Cuernavaca, Mexico; 4 Center for Tropical Diseases, University of Texas-Medical Branch, Galveston, Texas, United States of America; 5 Organización Latinoamericana para el Fomento de la Investigación en Salud, Bucaramanga, Colombia; 6 Hospital Infantil de México Federico Gómez, Mexico City, Mexico; 7 Duke-NUS Graduate Medical School, Singapore; 8 Pedro Kourí Tropical Medicine Institute, Havana, Cuba; 9 Pediatric Dengue Vaccine Initiative, Rockville, Maryland, United States of America; 10 University of California, Berkeley, Berkeley, California, United States of America; 11 Ministry of Health, Mexico City, Mexico; Oswaldo Cruz Foundation, BRAZIL

## Abstract

**Background:**

Dengue imposes a substantial economic and disease burden in most tropical and subtropical countries. Dengue incidence and severity have dramatically increased in Mexico during the past decades. Having objective and comparable estimates of the economic burden of dengue is essential to inform health policy, increase disease awareness, and assess the impact of dengue prevention and control technologies.

**Methods and Findings:**

We estimated the annual economic and disease burden of dengue in Mexico for the years 2010–2011. We merged multiple data sources, including a prospective cohort study; patient interviews and macro-costing from major hospitals; surveillance, budget, and health data from the Ministry of Health; WHO cost estimates; and available literature. We conducted a probabilistic sensitivity analysis using Monte Carlo simulations to derive 95% certainty levels (CL) for our estimates. Results suggest that Mexico had about 139,000 (95%CL: 128,000–253,000) symptomatic and 119 (95%CL: 75–171) fatal dengue episodes annually on average (2010–2011), compared to an average of 30,941 symptomatic and 59 fatal dengue episodes reported. The annual cost, including surveillance and vector control, was US$170 (95%CL: 151–292) million, or $1.56 (95%CL: 1.38–2.68) per capita, comparable to other countries in the region. Of this, $87 (95%CL: 87–209) million or $0.80 per capita (95%CL: 0.62–1.12) corresponds to illness. Annual disease burden averaged 65 (95%CL: 36–99) disability-adjusted life years (DALYs) per million population. Inclusion of long-term sequelae, co-morbidities, impact on tourism, and health system disruption during outbreaks would further increase estimated economic and disease burden.

**Conclusion:**

With this study, Mexico joins Panama, Puerto Rico, Nicaragua, and Thailand as the only countries or areas worldwide with comprehensive (illness and preventive) empirical estimates of dengue burden. Burden varies annually; during an outbreak, dengue burden may be significantly higher than that of the pre-vaccine level of rotavirus diarrhea. In sum, Mexico’s potential economic benefits from dengue control would be substantial.

## Author Summary

During the past decades, dengue fever has become the most common arthropod-borne viral disease, imposing a substantial economic and disease burden in most tropical and subtropical countries, including Mexico. Dengue incidence and severity have dramatically increased in Mexico, with transmission regularly reported in 28 of 32 states. Objective estimates of the burden of dengue are important to inform policy decisions and priorities. We merged multiple data sources to estimate (i) total episodes, (ii) costs per episode, (iii) surveillance and vector control costs, and (iv) disease burden (2010–2011). Results suggest that Mexico had about 139,000 symptomatic and 119 fatal dengue episodes per year on average. The annual cost, including surveillance and vector control, was about US$170 million, or $1.56 per capita, comparable to other countries in the Americas. Annual disease burden averaged 65 disability-adjusted life years per million population, with most of the years lost to disability corresponding to ambulatory episodes. The results show a substantial burden of dengue on the health care system and economy of Mexico. This quantification of the economic burden should help public health officials make informed decisions about current and promising new preventive and control measures to reduce dengue infections.

## Introduction

Dengue fever is the most important arthropod-borne viral disease affecting humans, with about half the world’s population estimated to be at risk of infection, and epidemics increasing in frequency, magnitude, and geographical reach [1–4]. Dengue imposes a substantial economic and disease burden in most tropical and subtropical countries. Mexico is no exception [5]. Dengue is hyperendemic in Mexico, with all four dengue virus (DENV) serotypes isolated in the country, high levels of disease and an increasing impact during the last decades [5–8]. Transmission of dengue is regularly reported in 28 of the 32 Mexican states; the main mosquito vector, *Aedes aegypti*, has been reported in 30 states [6,8,9]. The severity of dengue episodes has also steadily increased, with a substantial increase in severe dengue episodes since 1995, although case fatality rate has remained relatively low compared to other Latin American countries [7,10,11].

Objective, comparable measures of the burden of dengue are important to inform decisions about health policy, research, and health service priorities and to increase scientific and social awareness of the disease [12–15]. Despite the need for timely and reliable epidemiological data, dengue burden estimates are sparse. The total burden imposed by a disease includes the illness or disease burden, which measures the impact of a disease on morbidity and mortality in a specific population, and the economic burden [16,17], which includes the cost of illness, prevention and monitoring or surveillance strategies, and other economic impacts (e.g., decrease in travel, seasonal overload of health systems) [18,19]. Because dengue is a reportable illness in most endemic countries, an initial approximation of the total number of dengue episodes in a year is simply the total episodes reported to the country’s Ministry of Health (MoH) through surveillance systems.

Dengue is a reportable disease in Mexico; the MoH has promulgated protocols for laboratory confirmation and collects and disseminates weekly surveillance data [20]. The MoH is responsible for setting national guidelines, rules, and procedures that the 32 state health departments need to follow, although state and local health services are responsible for daily operations. Vector control and dengue surveillance systems guidelines are defined by the MoH at the federal level, although it collaborates with the 32 state health services and other health organizations including Mexican Institute of Social Security (IMSS), Institute of Social Security and Services for State Workers (ISSSTE), Mexican Petroleum (PEMEX), and the Armed Forces medical services [9,20]. A sample of patients with suspected DENV infection is diagnosed by a public health laboratory network (all probable patients in areas with no recent dengue episodes or during low transmission periods and about 30% of patients when there is evidence of transmission and during outbreaks) using confirmatory assays (NS1, IgM, or IgG ELISA), and a subset of these samples is analyzed for virus isolation (10% of the positive samples) [8,9,21]. Patients with probable and confirmed dengue have to be reported weekly, while probable or confirmed DHF and dengue-related deaths must be reported within 24 hours [20]. The MoH estimates the number of dengue episodes in two steps: probable cases are first multiplied by the proportion of positive cases from the lab-diagnosed sample (called possible cases), and then added to the total lab-confirmed cases. The MoH assumes that all episodes are notified [8].

However, passive surveillance systems have limitations. Passive surveillance systems are adequate for monitoring general trends in DENV infections; however, they usually underreport the total episodes of symptomatic dengue [22–26]. Febrile DENV infections with relatively mild symptoms have very low reporting ratios (number of reported dengue episodes / total dengue episodes in the population), and reporting increases with severity [27,28]. Other limitations in passive surveillance systems, even in well-funded systems such as Mexico or Puerto Rico, include misdiagnosis due to limited sensitivity of diagnostic tests, cost constraints, unrecognized dengue symptoms, variation in reporting ratios by severity of symptoms, and differences in diagnosis between epidemic and non-epidemic years [22,23,29–32]. Some health-seeking behaviors also reduce reporting ratios, such as symptomatic patients visiting alternative health providers, including pharmacies or local healers, or simply staying at home. In Mexico, there is little or no reporting from the private sector [33], and there is wide variation in the quality of reporting of notified cases. Limited reporting of symptomatic DENV infections leads to conservative estimates of economic and disease burden [24–26,34], which may affect health policy decisions. Many dengue-endemic countries are transitioning to the revised WHO dengue case classification [35]; however, while the new WHO classification is used in some clinical settings in Mexico, surveillance data are still compiled as DF and DHF [36].

The new web-based Epidemiological Surveillance Platform (EPS) was implemented in 2008 [8] in which health care workers enter cases directly into the national data base. It provides real-time data to support public health decisions. Before the EPS, dengue was reported in paper forms to state epidemiological departments, entered into a local electronic system, and then emailed to the federal health authority, which many times resulted in fragmented, non-compatible data [9]. Although the EPS has improved the quality of reporting, there is still substantial room for improvement: about 40% of the reports of dengue episodes in 2009 were still considered of bad or very bad quality [8]. We overcame this limitation adjusting officially reported dengue episodes, based on reporting ratios from a prospective cohort study in Morelos, Mexico, to obtain the overall number of symptomatic DENV infections.

In addition to surveillance strategies, prevention and control are a substantial part of the economic burden of dengue. While there are various promising dengue prevention and control technologies under development [37–40], currently the only way to prevent DENV transmission is to control the vector population [41]. Vector control, prevention, and surveillance are financed through the MoH at the federal level, including the design and maintenance of the EPS. Prevention and control activities include entomological surveillance and risk assessment through mosquito ovitraps and larval indices, as well as vector control activities such as insecticide nebulization, indoor spraying, and use of larvicides. Other activities include educational and awareness campaigns, training health and vector control personnel, and community-based participatory control programs [9,33,42,43].

The objective of this study was to measure the economic and disease burden of symptomatic DENV infections in Mexico. We estimated the economic costs of dengue using a societal perspective, including vector control and surveillance costs, and the disease burden of dengue in disability-adjusted life-years (DALYs). Previous studies have estimated the economic and disease burden of dengue illness in countries from the Americas [44–52], including Mexico [44]. However, these estimates for Mexico are limited due to incomplete data and extrapolation from neighboring countries [53]. Here we addressed these limitations by combining data from multiple sources and refined estimates of the economic and disease burden of dengue in Mexico. Specifically, we estimated (i) total average annual number of dengue episodes, (ii) unit costs per episode, (iii) vector control and surveillance costs, and (iv) disease burden using DALYs. With this study, Mexico joins Panama [45], Puerto Rico [47], Nicaragua [49], and Thailand [54] as the only countries or areas worldwide with comprehensive (illness and preventive) peer-reviewed empirical estimates of the cost of dengue.

## Materials and Methods

### Overview

We estimated the economic burden of dengue from a societal perspective and the disease burden of dengue in DALYs, using the WHO methodology [55,56]. Specifically, we used the following equations:

Economicburdenofdengue(USdollars)=totalepisodesxcostsperepisode+denguepreventionandsurveillanceactivities+othereconomicimpacts

Diseaseburdenofdengue(DALYs)=yearsoflifelost(YLL)duetoprematuredeath+yearslivedwithdisability(YLD)

An accurate estimate of the total number of dengue episodes is critical to obtain the economic and disease burden of dengue, and previous studies have found that uncertainty in the total number of dengue episodes is the main source of variability [44,57]. The costs per dengue episode include direct medical and non-medical costs and indirect costs per non-fatal and fatal case. The burden of disease was measured in DALYs, a summary measure of population health that combines information on mortality and non-fatal disease outcomes [16].

We based our burden estimates on the years 2010 and 2011 because we had access to detailed surveillance data in those years from the EPS [8,9]. The years 2010 and 2011 are, on average, relatively close to historical averages in reported cases. The average annual reported episodes were 30,941 in 2010–2011, 58,688 in 2007–2011 (which includes the 2009 outbreak), 35,091 in 2002–2011, and 32,886 in 1995–2011 [8]. Thus, if anything, our burden estimates are slightly conservative considering long-term patterns. Last, we performed a probabilistic sensitivity analysis of the economic and disease burden estimates using Monte-Carlo simulations. Monte Carlo simulations are commonly used to model phenomena with substantial uncertainty in its parameters. The method relies on running repeated trials based on random sampling from the probability distribution of each parameter in the model, and recording the results of each simulation. The results from the repeated trials were used to describe the uncertainty in the model. We report our results in 2012 US dollars (USD) using the 2012 exchange rate (USD1 = 12.88 Mexican pesos, MXN), and GDP deflators [58].

### Number of dengue episodes

To refine the estimates of the total number of dengue episodes, officially reported dengue episodes can be adjusted for underreporting using an expansion factor (EF). An EF can be calculated as the analyst’s best estimate of the total number of dengue cases in a population divided by the number of reported cases considered dengue (EF = 1/reporting ratio) [59]. We estimated total episodes of dengue by multiplying reported episodes (41,333 episodes in 2010, and 20,548 in 2011) by an empirical EF derived from a prospective cohort study in Morelos [60,61].

The prospective cohort study was conducted in a dengue-endemic urban area in Morelos, Mexico, to assess the rate of DENV infections among the neighbors of reported dengue cases [61]. Set in the towns of Tepalcingo and Axochiapan during the 2011–2012 dengue season (June 2011-March, 2012), the study contained 1,172 participants aged 5 years and above. All participants or the parent or legal guardian of minors (5–17 years of age) gave written informed consent. The Morelos study was approved by the Ethics Commission of the National Institute of Public Health, Mexico and the Brandeis University Committee for Protection of Human Subjects.

Researchers collected 10-ml blood samples (6-ml for serological diagnosis and 4-ml for DNA extraction [62]) from all participants at baseline and 6-ml in a follow-up 3–4 months later, in addition to demographic, environmental, health-seeking behavior (e.g., number of visits to health care facilities, type of facility, private or public), and socio-cultural and entomological data. Passive and active monitoring occurred between the two rounds of data collection, including phone calls or house visits at least once a month. All dengue episodes were laboratory-confirmed by means of a paired DENV-specific IgM and IgG Capture ELISA (PanBio) at baseline and follow-up. Recent DENV infections were defined as: (i) IgM or IgG positive by capture assay, which measures recent dengue infection (2–3 months) in the baseline sample (pre-enrollment infections)[63,64], (ii) IgM or IgG positive in the follow-up sample where IgM and IgG were negative in the baseline sample (post-enrollment infections), and (iii) availability of RT-PCR/NS1/IgM/IgG positive during the follow-up months from a visit to the local health service. We used the blood samples collected at baseline or follow-up to confirm DENV infection; 12 patients were also diagnosed during the febrile episode by the state of Morelos health services (Servicio de Salud de Morelos) using NS1 or IgM/IgG capture assays. Symptomatic dengue episodes were defined as lab-confirmed dengue and reported fever.

Morelos provides a good reference value of reporting ratios of dengue episodes in Mexico. Morelos has strengthened its epidemiological surveillance in recent years, there is high level of dengue awareness and willingness to participate in dengue surveillance among the population and clinicians in the public health sector [33]. A recent study of benchmarking of effective healthcare coverage (“the proportion of potential health gain that could be delivered by the health system to that which is actually delivered”, p.1729) in Mexico based on 14 healthcare interventions [65], suggests that Morelos’ quality of healthcare provision is not too different from the country’s average. Specifically, compared to other states Morelos’ measure of effective coverage was 0.54 standard deviations below the national mean or at the 30^th^ percentile nationally. Recent studies have used healthcare indicators to estimate reporting ratios of dengue, based on access [16] and quality [34] of healthcare, with the latter probably better reflecting the idiosyncrasies of the system that may lead to underreporting. For these reasons, we consider that using the Morelos prospective cohort study to obtain point estimates of dengue burden is reasonable, and if anything, slightly conservative. To adjust for variation in reporting ratios, we used empirical estimates of EFs from a previous study of dengue in the Americas [44] in the sensitivity analysis.

### Economic burden of dengue

We derived costs per episode by combining patient interviews in four major hospitals in the states of Quintana Roo, Morelos, and Tabasco, macro-costing data from two major public hospitals in Tabasco, MoH health and surveillance data [66], WHO-CHOICE [67] estimates for Mexico, and previous literature on dengue burden. Indirect costs were obtained based on productivity losses by age, considering both the patient and the patient’s caregivers. We estimated vector control and surveillance costs based on MoH data.

**Direct costs per episode.** We estimated unit costs per dengue bed-day (inpatient episodes) and per visit (outpatient episodes) by combining macro-costing data from two major public hospitals, MoH surveillance data [66], WHO-Choice estimates [67], data from the Morelos cohort study [60], and national health statistics [66,68]. Direct medical unit costs were obtained using a macro-costing technique based on data reported by two tertiary public hospitals. To derive direct medical inpatient and outpatient unit costs that were representative of the country, we derived cost ratios for the treatment of dengue in various settings from WHO-CHOICE costs estimates for Mexico [67]. For hospitalized patients, we estimated the relative weight of treated episodes in each type of hospital based on its share total hospital beds (obtained from national health statistics) assuming that the proportion of patients who are treated in each type of hospital is equal to its fraction of total hospital beds. For ambulatory episodes, we obtained the relative weights of episodes treated in each type of setting by combining data from the Morelos cohort study (share of patients who did not visit a private or public health facility), average annual outpatients visits from health statistics and WHO-CHOICE estimates (used a proxy for its relative utilization) [67,68].

As the study did not provide any treatment to participants, it was unlikely to have any major effect on health care utilization. While receiving regular questions about febrile illness may have sensitized participants through a Hawthorne effect [69], we expect the effect to be small, if any, since there was already substantial awareness of dengue in the area [33].

The costs for homecare (including pharmacy visits) were derived from combining data sources. The share of patients with apparent dengue who did not visit a hospital or health center (about 30%, largely consistent with a previous study of healthcare use [70]) were obtained from the Morelos cohort study. Of those patients who did not visit a health center, about 37% visited a pharmacy at the onset of their febrile illness. We derived the average expenditures on medications, transport, and diagnostic tests of these early pharmacy visits from36 interviews of hospitalized dengue patients (S1 Table). We assumed that the patients who stayed at home had similar costs in medications as those who visited a pharmacy at the onset of their illness, but no transport or diagnostic costs associated with their dengue episode. The hospitalized patient interviews were also used to obtain non-medical direct costs, including transport, food, and hotel expenditures for dengue patients who visited a healthcare facility and their caregivers (S1 Text).

**Fig 1:**
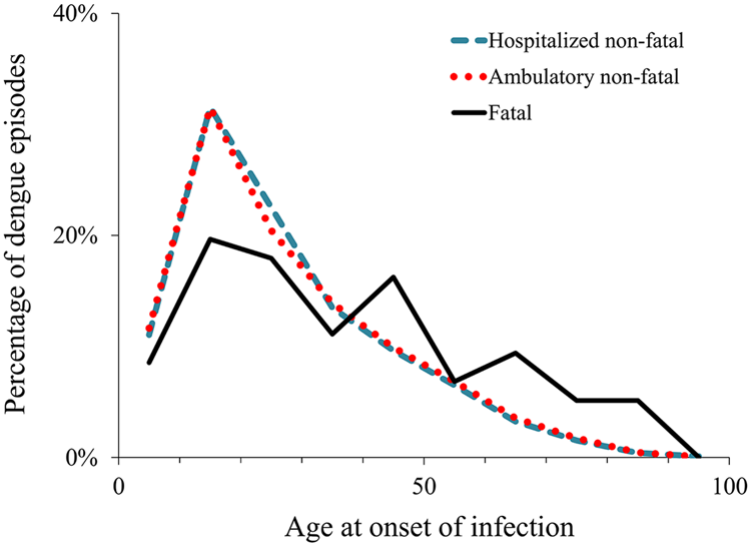
Age distribution of reported dengue episodes, 2010–2011. Notes: The graph shows the percentage of reported episodes in each 10-year age category, from 0–9 through 90–99, using the midpoint of each category.

**Indirect costs per episode.** We used the human capital approach, based on work-time loss caused by dengue, to derive indirect costs per fatal and non-fatal episode [71]. Productivity loss estimates included days of work or school lost by the patient as well as relatives’ time spent caring for the patient. The breakdown by age and occupation at onset of dengue illness affect the estimates of productivity loss. Fig. 1 shows the breakdown by age of the reported cases in years 2010 and 2011. The breakdown by occupation was derived assuming that all patients aged 5–15 years old were enrolled in school, patients aged 16–17 were divided between school and work based on empirical data from school enrollment [72]. We derived the average economic value of a work day lost for economically active adults (employed or actively looking for employment) based on Mexico’s wage distribution and employment rate from the Mexican National Institute of Statistics and Geography for patients aged over 17 years old [73]. For non-economically active adults (unemployed and not actively looking for employment), the estimate was based on their reported main activity (students, household chores, retired, disabled, and non-active).

Individual and societal costs of school absence are difficult to value, but, being conservative, are at least equal to the cost of providing a day of school. We derived unit cost per-day of school lost using data on total educational expenditure at the federal, state, and municipal levels, and from the private sector, for years 2010 and 2011. A school year averages 200 schools days [74].

Economic loss from days lost was valued as the number of days lost to dengue illness times the average value per day. We took the length of hospitalization from the Mexican MoH surveillance data. We estimated the durations of illness for ambulatory and hospitalized patients as the average values from surveys of dengue patients in the corresponding setting (326 hospitalized and 834 ambulatory) across five countries in the Americas (Brazil, El Salvador, Guatemala, Panama, and Venezuela) [46]. We obtained the number ambulatory visits of hospitalized patients from interviews with them or their caregivers in Mexico. For ambulatory episodes, we used the same surveys from the Americas to derive the average duration of illness and total healthcare visits [46]. We based indirect costs of fatal cases on productivity losses by age using the age distribution of reported deaths from MoH surveillance data, the average economic value of a work day (see above), and a 3% discount rate (for consistency with international recommendations and previous studies) [71]. We estimated the years of premature life lost based on life expectancy using WHO life tables [75]. Due to the paucity of data, we assumed that the rate of reporting of deaths attributed to dengue was equal to the rate of reporting in hospitalized episodes. We relaxed this assumption in the sensitivity analysis.

**Dengue prevention and surveillance.** We estimated vector control and surveillance costs based on the Mexican MoH annual budget for dengue. The available data included only the years 2009 and 2010, so we imputed the vector and surveillance budget in 2011, using the average budget of the two previous years with adjustment for inflation. While vector control and dengue surveillance systems are managed by the MoH at the federal level, our estimates are conservative as they do not include possible additional spending on surveillance and vector control by state level agencies and municipalities (mainly nebulization, larvae control, and patio clean-up campaigns).

**Other economic impacts of dengue.** While important, data limitations did not allow us to reasonably estimate other economic costs associated to symptomatic DENV infections. These impacts include the detrimental effect of dengue outbreaks on tourism and travel [76–79], co-morbidities and complications associated with dengue infection [80–85], or the effects of health system overload [86]. When dengue outbreaks are clustered in time or location [87–90], they may worsen treatment quality and decisions or degrade performance of clinical laboratories.

### Dengue burden of disease

The burden of disease was measured in DALYs, and is composed of the person’s years of life lost (YLL) due to premature death—based on incidence, fatality rate, and life expectancy, and a measure of the time the person lives in less than full health (years lived with disability, YLD)—based on incidence, length of illness, and impact on quality of life [14]. We estimated the burden of disease using the WHO methodology [55,56], for comparability with previous studies, and expressed burden in DALYs per million population. We obtained the age distribution of non-fatal dengue episodes and deaths from surveillance data (Fig. 1) and used model parameters (age weights, disability weight, and discount rate) based on previous studies [44,52,91]. Because the 2010 estimates of global burden of disease [16] changed their definition of DALYs (dropping age weighting and time discounting), we also provide these numbers in the results section for comparability with future estimates. Under this new definition, a child death converts to a larger number of DALYs than previously.

### Sensitivity analysis

We used a probabilistic sensitivity analysis to address the uncertainty in our estimates of the disease and economic burden of dengue. We computed 10,000 Monte Carlo simulations simultaneously varying our parameter estimates for EFs, unit costs, days lost per episode, health service utilization, and household impact using RiskAMP [92] (iterations drew random values from the distribution of each input using the Mersenne Twister random number generator). Our results include a base-case scenario, using our best estimates for each parameter, the uncertainty around these estimates based on the sensitivity analyses, and 95% certainty level (CL) bounds.

To model variation in reporting ratios, we used a beta-PERT distribution (hereafter PERT) with the Morelos cohort empirically-derived EF as our best estimates. The range of variation in the distribution was based on a recent study of dengue in the Americas [44], which identified five field studies that included reporting ratios. We used conservative estimates, including an EF of 1.0 as the lower bound in hospitalized cases. We also used a PERT distribution for direct medical costs, with the minimum and maximum values obtained in primary and tertiary hospitals for hospitalized cases and homecare and tertiary hospitals for ambulatory episodes from combining WHO-CHOICE [67] estimates, health statistics data [66,68], macro-costing estimates, expert opinion, and patient interviews. We derived direct non-medical costs from patient interviews in four major hospitals. The variation in duration of dengue episodes and health service utilization was estimated using a normal distribution with parameters based on detailed MoH surveillance data, hospital interviews, and empirical estimates from a previous study in five countries in the Americas [46]. Last, we used a normal distribution of household impact based on weighted averages from Suaya et al.[46]

## Results

### Input for the estimation model

**Expansion factors to adjust reported episodes.** Table 1 shows a summary of the results from the prospective cohort study in Morelos, Mexico. We found a total of 253 DENV infections. Most of these infections were asymptomatic (61%), consistent with previous studies [93–96]. Only 67% of the participants with symptomatic infections visited a doctor, and most of them (74%) sought care at least once in the public sector. Of all symptomatic dengue episodes that were attended by a doctor either as outpatient or inpatient, 32% were reported to the State of Morelos surveillance system [60]. In other words, for every symptomatic episode of dengue that was treated by a health professional and reported to the surveillance system, 3.1 episodes occurred. If we considered all cases of symptomatic dengue, irrespective of whether they were attended by a healthcare professional or not, only 21% were reported to the surveillance system. Thus, there were 4.7 symptomatic dengue episodes for every reported symptomatic episode.

**Table 1:** Main results from the prospective cohort study in Morelos, Mexico 2011–2012.

Town	Participants	Total	Symptom.	Visited	Public facility ^a^			Private facility only		
	(n)	infections	infection	a facility	Rep.	Not rep.	Total	Rep.	Not rep.	Total
**Pre-enrollment infections (up to 2–3 months before enrollment)**										
*Tepalcingo*	*386*	*49*	*18*	*12*	*4*	*6*	*10*	*0*	*2*	*2*
Ambulatory				11	3	6	9	0	2	2
Hospitalized				1	1	0	1	0	0	0
*Axochiapan*	*786*	*164*	*65*	*40*	*15*	*15*	*30*	*0*	*10*	*10*
Ambulatory				36	12	14	26	0	10	10
Hospitalized				4	3	1	4	0	0	0
**Sub-total**	**1,172**	**213**	**83**	**52**	**19**	**21**	**40**	**0**	**12**	**12**
**Post-enrollment infections**										
*Tepalcingo*	*318*	*8*	*1*	*1*	*1*	*0*	*1*	*0*	*0*	*0*
Ambulatory				0	0	0	0	0	0	0
Hospitalized				1	1	0	1	0	0	0
*Axochiapan*	*604*	*32*	*15*	*13* ^*b*^	*1*	*7*	*8*	*0*	*5*	*5*
Ambulatory				12	1	6	7	0	5	5
Hospitalized				1	0	1	1	0	0	0
**Sub-total**	**922**	**40**	**16**	**14**	**2**	**7**	**9**	**0**	**5**	**5**
***Total***	***1*,*172***	***253***	***99***	***66***	***21***	***28***	***49***	***0***	***17***	***17***

Notes: Rep. denotes case reported through the surveillance system. Subtotals by municipality are italicized.

^a^ Includes patients who only visited public healthcare sites and who visited both private and public sites.

^b^ For one person we do not know whether she/he visited a health facility.

Reporting ratios vary considerably between the public and private sectors. In the public sector, 43% of the dengue episodes were reported (reporting ratio = 0.43), whereas no dengue episode was reported by the private sector. Limited or no reporting from the private sector has also been noted elsewhere [27,33,97]. Based on these results, Table 2 shows a summary of the expansion factors for overall (EF_T_), hospitalized (EF_H_), and ambulatory (EF_A_) dengue episodes needed to estimate the total cases of symptomatic dengue.

**Table 2:** Summary expansion factors (EF) for symptomatic DENV infections, based on the Morelos prospective cohort study (2011–2012).

Setting	Dengue patients who visited a health facility	All symptomatic DENV infections
Ambulatory	3.7	5.6
Hospitalized	1.4	2.0
Total	3.1	4.7

Notes: Rep. denotes case reported through the surveillance system. DENV denotes dengue virus. Expansion factors weighted by total episodes in each category.

**Direct medical and non-medical unit costs.** Table 3 shows the estimation procedure and main data used to obtain direct medical unit costs using macro-costing [98]. Combining these data with the distribution of cases and the cost ratio relative to a tertiary hospital, we derived an average cost estimate per bed-day ($240.04) and per outpatient visit ($65.53), as shown in Table 4.

**Table 3:** Summary of direct medical unit costs (2012 US dollars) derived using macro-costing with data from two tertiary hospitals in Tabasco.

Row	Item	Source	Hospital 1	Hospital 2
(1)	Number of registered beds (official)	Reported by hospital	*153*	*206*
(2)	Average occupancy rate	Reported by hospital	*97%*	*67%*
(3)	Occupied beds	*(1) × (2)*	148	137
(4)	Annual bed-days	*(3) × 365*	54,002	50,152
(5)	Total ambulatory visits	Reported by hospital	*109*,*612*	*134*,*073*
(6)	Relative cost: outpatient visit/inpatient day	Shepard et al.[98]	0.32	0.32
(7)	Ambulatory bed-day equivalents	*(5) × (6)*	35,076	42,903
(8)	Total bed-day equivalents	*(4) + (7)*	89,078	93,055
(9)	Total hospital operating expenditures, $	Reported by hospital	*24*,*528*,*597*	*28*,*981*,*402*
(10)	Cost per bed-day equivalent, $	*(9) / (8)*	275.36	311.44
(11)	Cost per ambulatory visit, $	*(10) × (6)*	88.12	99.66
(12)	GNP per capita, $	World Bank	10,064	10,064
(13)	Bed-day as share of GNP per capita	(10) / (12)	*2*.*74%*	*3*.*09%*

Notes: The numbers in *italics* were reported by the hospitals. Costs correspond to year 2011 and were adjusted to 2012 US dollars, using gross domestic product (GDP) deflators [58]. Operating expenditures include personnel costs, administrative services and equipment, drugs, exams and other medical supplies, maintenance and new medical equipment, maintenance and acquisition of vehicles and buildings, and utilities. GNP denotes gross national product.

**Table 4:** Estimation of direct medical unit costs (2012 US dollars) per bed-day and outpatient visit to a public hospital.

Item and type of hospital	Distribution of cases (%)	Ratio of cost to tertiary hospital ^a^	Unit costs per type of facility	Best estimate, unit costs
*Per bed-day* ^*b*^				$ 240.04
Primary-level hospital	65%	0.74	$ 230.54	
Secondary-level hospital	26%	0.77	$ 240.52	
Tertiary-level hospital	9%	1.00	$ 311.01	
*Ambulatory visit* ^*c*^				$ 65.53
Homecare or pharmacy	30%	0.17	$ 17.23	
Health center (no beds)	19%	0.68	$ 67.87	
Primary-level hospital	13%	0.84	$ 83.82	
Secondary-level hospital	27%	0.96	$ 95.50	
Tertiary-level hospital	11%	1.00	$ 99.52	

Notes: ^a^ The ratio between the costs of each type of hospital compared to a tertiary hospital in Mexico was derived from the WHO-Choice estimates for Mexico [67].

^*b*^ The distribution of dengue episodes by type of setting was estimated as proportional to the number of beds by setting for hospitalized cases based on MoH health statistics [66].

^*c*^ For ambulatory visits, we obtained the distribution and costs of dengue episodes by type of setting combining data from the Morelos cohort study, hospital questionnaires, expert opinion, WHO estimates, and MoH data. The share of patients that did not seek healthcare (30%) was obtained from interviews in the Morelos cohort study. This estimate is consistent with a 70% probability of using paid or unpaid healthcare services in Mexico obtained by Dávila and Guijarro (2000) [70] using the National Household Income and Expenditure Survey. Patients who sought care were distributed by type of facility based on average annual outpatients visits [68], and WHO-CHOICE estimates [67].

Non-medical direct costs were obtained from patient interviews. For hospitalized patients, daily non-medical costs were $25.16 for adults and $27.85 for children, and daily non-medical costs for ambulatory patients were $11.96 for adults and $9.09 for children, on average. For hospitalized patients, additional daily non-medical costs for other household members were $8.39 for adults and $6.56 for children. For ambulatory patients, the additional daily non-medical costs for other household members were $3.00 for adults and $6.00 for children.

**Indirect unit costs.** We estimated indirect costs based on productivity loss from the number of school-days and work-days lost. The estimated average daily unit costs for elementary education (5–14 year olds) were $7.32 in 2010, and $7.59 in 2011, and for high school education (15–18 year olds) were $9.05 in 2010 and $9.14 in 2011 [72,99]. A work-day lost for economically active adults was estimated at $10.93/day in 2010 and $11.06/day in 2011 and for non-economically active adults at $4.26 in 2010 and $4.22 in 2011. Overall, the economic value of the average work day lost was $8.20 in 2010 and $8.22 in 2011; about 1.7 times the minimum wage, which is consistent with estimates from previous studies [46,97].

**Duration of dengue episodes and productivity loss.** We estimated the duration of hospitalized dengue episodes at 13.9 days, including both the acute and the convalescent phases (7.4 days acute phase, 6.5 days convalescent phase). Based on hospital interviews in Mexico, we estimated that an adult had 2.4 ambulatory visits on average before being hospitalized, and a child had an average of 3.7 ambulatory visits prior to hospitalization. Ambulatory patients had a total of 3.9 healthcare visits, and illness had a total duration of 12.0 days. From the interviews, we obtained that each hospitalized patient affected on average 1.7 adults and 0.6 children in the household, and each ambulatory patient affected 2.2 adults and 0.4 children in the household on average. At the household level, school-days lost were 3.7 days for inpatients and 2.2 days for outpatients, and 6.1 work-days were lost for inpatients and 3.8 work-days were lost for outpatients.

**Summary of parameters and probability distributions for sensitivity analysis.** Table 5 shows a summary of the main parameters used in the analysis, assumed probability distributions, and sources. The parameters described above were used to derive base case point estimates, and the distributions and range were used in the sensitivity analysis to obtain 95% certainty levels of economic and disease burden (show in parentheses in the tables henceforth).

**Table 5:** Summary of the parameters varied simultaneously in the sensitivity analysis, assumed probability distributions, and data sources.

Item	Units	Estimate	Distribution	Statistics	Value	Source
*(1) Expansion Factors*						
Hospitalized	EF_H_	2.0	PERT	(Min; Best; Max)	(1.0; 2.0; 3.3)	Morelos cohort, Shepard et al.[44]
Ambulatory	EF_A_	5.6	PERT	(Min; Best; Max)	(5.0; 5.6; 15.0)	Morelos cohort, Shepard et al.[44]
*(2) Direct medical costs*						
Hospitalized	$	238.91	PERT	(Min; Best; Max)	(229.5; 238.9; 309.5)	Macro-costing, WHO [67], MoH [66]
Ambulatory	$	65.25	PERT	(Min; Best; Max)	(17.23; 65.3; 99.1)	Macro-costing, WHO [67], MoH [66,68], Morelos cohort, interviews.
*(3) Direct non-medical costs*						
Hospitalized-adults	$	25.16	Normal	(μ, σ)	(25.2; 7.0)	Patient interviews
Ambulatory-adults	$	11.96	Normal	(μ, σ)	(12.0; 8.3)	Patient interviews
Hospitalized-children	$	27.85	Normal	(μ, σ)	(27.8; 6.4)	Patient interviews
Ambulatory-children	$	9.09	Normal	(μ, σ)	(9.1; 2.0)	Patient interviews
*(4) Duration of episode (acute + convalescent phase)*						
Hospitalized	Days	13.9	Normal	(μ, σ)	(13.9; 5.3)	MoH surveillance, Suaya et al.[46]
Ambulatory	Days	12.3	Normal	(μ, σ)	(12.3; 5.4)	MoH surveillance, Suaya et al.[46]
*(5) Health service utilization*						
Hospitalized	Days	3.5	Normal	(μ, σ)	(3.5; 4.3)	MoH surveillance
Amb. (pre-hospital, adult)	Days	2.4	Normal	(μ, σ)	(2.4; 1.1)	Patient interviews
Amb. (pre-hospital, child)	Days	3.7	Normal	(μ, σ)	(3.7; 2.5)	Patient interviews
Ambulatory	Days	3.9	Normal	(μ, σ)	(3.9; 2.1)	Suaya et al.[46]^*a*^
*(6) Patient impact (average days lost by patient)*						
Hospitalized, school loss	Days	6.2	Normal	(μ, σ)	(6.2; 4.2)	Suaya et al.[46]^*a*^
Ambulatory, school loss	Days	4.4	Normal	(μ, σ)	(4.4; 3.3)	Suaya et al.[46]^*a*^
Hospitalized, work loss	Days	9.8	Normal	(μ, σ)	(9.8; 4.3)	Suaya et al.[46]^*a*^
Ambulatory, work loss	Days	5.4	Normal	(μ, σ)	(5.4; 4.3)	Suaya et al.[46]^*a*^
*(7) Household impact (average days lost by each household member affected)*						
Hospitalized, school loss	Days	3.7	Normal	(μ, σ)	(3.7; 4.5)	Suaya et al.[46]^*a*^
Ambulatory, school loss	Days	2.2	Normal	(μ, σ)	(2.2; 3.5)	Suaya et al.[46]^*a*^
Hospitalized, work loss	Days	6.1	Normal	(μ, σ)	(6.1; 6.6)	Suaya et al.[46]^*a*^
Ambulatory, work loss	Days	3.8	Normal	(μ, σ)	(3.8; 5.3)	Suaya et al.[46]^*a*^

Notes: Normal distributions for medical expenditures and days lost were lower-truncated at zero. PERT (min, best, max, λ) with λ = 4. EF denotes expansion factor, WHO denotes World Health Organization, MoH denotes Ministry of Health.^*a*^ Simple average from countries from the Americas included in Suaya et al. [46] (Brazil, El Salvador, Guatemala, Panama, and Venezuela).

### Estimated economic and disease burden

**Total adjusted symptomatic DENV infections.** Table 6 shows a summary of reported cases by setting for years 2010 and 2011, and the total estimated cases using EFs. MoH reported episodes of dengue include lab-confirmed episodes plus the proportion of positive cases from the lab-diagnosed sample multiplied by the probable cases reported (probable dengue are suspected episodes of dengue with specific clinical symptoms). Overall, we estimated a total of 195,154 (95%CL: 180,459–355,343) non-fatal and 126 (95%CL: 80–180) fatal episodes of symptomatic dengue in 2010, and 82,429 (95%CL: 75,203–142,041) non-fatal and 112 (95%CL: 75–170) fatal episodes of dengue in 2011.

**Table 6:** Total symptomatic DENV infections reported by the Ministry of Health and estimated episodes using expansion factors from the Morelos cohort study.

	2010				2011^*a*^			
Data	DF	DHF	Deaths	Total	DF	DHF	Deaths	Total
*MoH estimated cases*								
Hospital	3,454	6,224		9,760	3,092	5,723		8,992
Ambulatory	29,396	2,200		31,574	11,063	654		11,556
Total	32,850	8,424	62	41,333	14,154	6,377	55	20,548
*Adjusted using EFs*								
Hospital	7,014	12,639		19,820	6,278	11,623		18,261
(95%CL)				(12,591–28,282)				(11,600–26,057)
Ambulatory	163,231	12,216		175,325	61,430	3,632		64,168
(95%CL)				(161,389–334,776)				(59,068–122,527)
Total	170,245	24,854	126	195,145	67,708	15,255	112	82,429
(95%CL)			(80–180)	(180,459–355,343)			(71–159)	(75,203–142,041)

Notes: Estimated cases from the Mexican Ministry of Health (MoH) include all lab-confirmed cases plus the share of positive cases from the laboratory diagnosed samples multiplied by the probable cases reported (probable dengue are suspected episodes of dengue with specific clinical symptoms). 95%CL denotes a 95% certainty level for each estimate, obtained through Monte Carlo simulations using the probability distributions shown in Table 5. These numbers in parentheses indicate the region of uncertainty around base-case estimates. DENV denotes dengue virus, DF denotes dengue fever, DHF denotes dengue hemorrhagic fever, EF denotes expansion factor, CL denotes certainty level.

^*a*^ In year 2011, 13.7% of the DF episodes and 3.1% of the DHF episodes reported by the MoH were not classified as ambulatory or hospitalized cases in the data. We assigned these patients to hospitalized or ambulatory treatments based on the proportion of patients by treatment in 2010.

**Fig 2:**
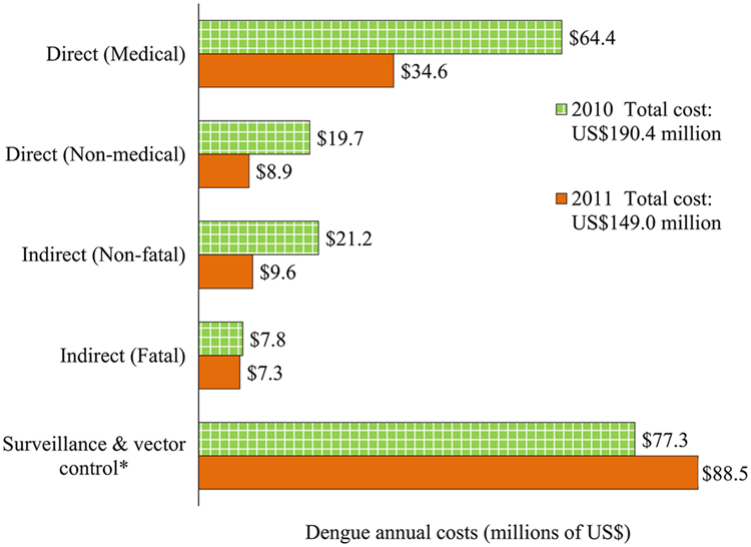
Distribution of the economic burden of dengue in Mexico by component, 2010–2011. Notes: Costs were adjusted to 2012 US dollars, using gross domestic product (GDP) deflators. [58] *Due to limited availability of data, vector control and surveillance costs for the year 2011 were estimated based on the average annual budget allocated by the Mexican Ministry of Health from the previous two years (2009 and 2010).

**Economic burden of dengue.** The average cost per non-fatal dengue episode was $1,327 for hospitalized patients (direct medical: $1,010; direct non-medical: $174; indirect: $143) and $451 for ambulatory patients (direct medical: $253; direct non-medical: $92; indirect: $106). The average indirect cost per fatal dengue episode was $63,817. Altogether, the aggregate economic cost of dengue was $190 (95% CL: $165-$357) million in 2010, with a per capita costs of $1.76 (95% CL: $1.52-$3.29), and $149 (95% CL: $136-$231) million in 2011 with $1.36 (95% CL: $1.24-$2.11) per capita (Table 7). These results amount to an average economic burden of dengue of $170 (95% CL: $151-$292) million, or $1.56 (95% CL: $1.38-$2.68) per capita. The fatal episodes of dengue represent a relatively small share of the total economic burden (4.5% on average for years 2010 and 2011). Surveillance and vector control cost about $0.76 per capita ($0.71 in 2010 and $0.81 in 2011), and this represents about 48.9% of the total economic burden of dengue in Mexico (Fig. 2).

**Table 7:** Economic burden for adjusted dengue episodes in Mexico (2012 US dollars), 2010–2011.

	2010		2011		Average 2010–2011	
	Cost (millions)	Per capita	Cost (millions)	Per capita	Cost (millions)	Per capita
Hospitalized	$26.39	$0.24	$24.15	$0.22	$25.27	$0.23
(95% CL)	(14.28–82.41)	(0.13–0.76)	(13.02–75.59)	(0.12–0.69)	(13.63–78.98)	(0.13–0.73)
Ambulatory	$78.93	$0.73	$28.96	$0.27	$53.95	$0.50
(95% CL)	(50.17–226.87)	(0.46–2.09)	(18.44–83.31)	(0.17–0.76)	(34.28–155.15)	(0.32–1.43)
Fatal	$7.82	$0.07	$7.32	$0.07	$7.57	$0.07
(95% CL)	(4.91–11.23)	(0.05–0.10)	(4.60–10.51)	(0.04–0.10)	(4.75–10.87)	(0.04–0.10)
Surv. & vector control	$77.3	$0.71	$88.54	$0.81	$82.92	$0.76
**Total**	$**190.45**	$**1.76**	$**148.97**	$**1.36**	$**169.71**	$**1.56**
**(95% CL)**	**(164.51–356.96)**	**(1.52–3.29)**	**(135.84–230.61)**	**(1.24–2.11)**	**(150.52–291.50)**	**(1.38–2.68)**

Notes: 95% CL denotes a 95% certainty level for each estimate, obtained through Monte Carlo simulations using the probability distributions shown in Table 5. These numbers in parentheses indicate the region of uncertainty around base-case estimates. Surv. & vector control denotes the costs of surveillance and vector control based on the Ministry of Health annual budget.

Fig. 2 shows the distribution of the economic burden of dengue in Mexico. Direct medical costs represent ~29% of the total average economic costs of dengue (34% in 2010; 23% in 2011), and direct non-medical costs sum ~8% of the total costs (10% in 2010; 6% in 2011). Fatal and non-fatal indirect costs, due to productivity loss, represent ~14% of the total economic costs of dengue (15% in 2010; 11% in 2011).

**Fig 3:**
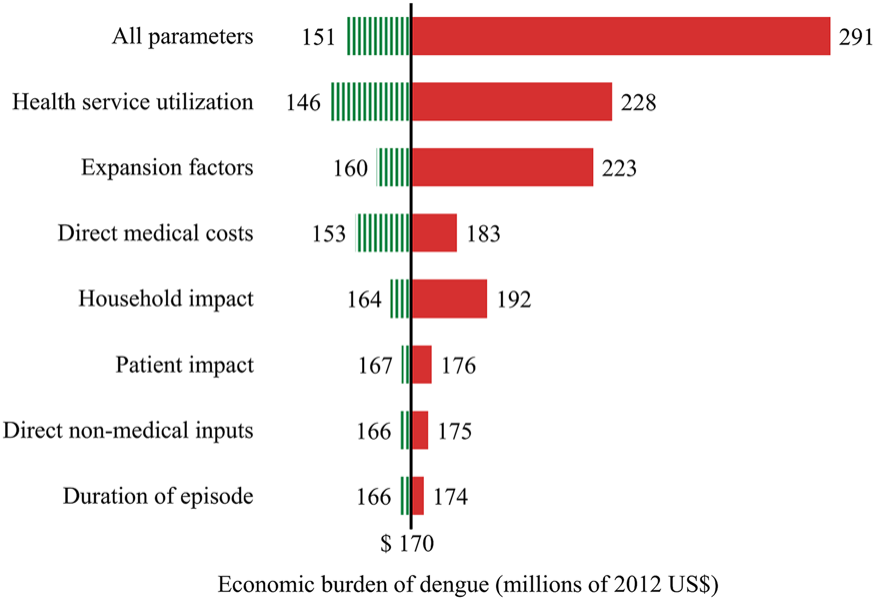
Variation in total economic burden of dengue based on listed parameters included in the sensitivity analysis (average for years 2010 and 2011). Notes: The vertical line shows the point estimate for the average total economic burden of dengue ($170 million). The variation for each parameter corresponds to the 95% certainty level obtained through the computation of 10,000 Monte Carlo simulations for each parameter, and for the simultaneous variation of all parameters (bar at the top). A summary of the main parameters, assumed distributions and data sources are shown in Table 5.

The main sources of variation for the economic burden of dengue estimates are shown in the tornado plot in Fig. 3. The vertical line shows the point estimate for the average total economic burden of dengue ($170 million). The variation for each parameter corresponds to the 95% certainty level obtained through the computation of 10,000 Monte Carlo simulations for each parameter, and for the simultaneous variation of all parameters (top bar). The diagram shows that health service utilization represents the biggest source of variation among the parameters considered in the sensitivity analysis in this study, closely followed by EFs to refine estimates of reported dengue episodes.

**Disease burden of dengue.** The total disease burden for the adjusted average of dengue episodes was 65.1 (95%CL: 36.0–98.7) DALYs per million population (83.5 in 2010; 46.7 in 2011). Fatal episodes represented about 27% of the disease burden of dengue (DALYs) in 2010 and 45% of the disease burden in 2011 (2010: 22.3 YLL; 2011: 20.8 YLL). The Institute of Health Metrics and Evaluation’s 2010 global disease burden study (GBD 2010) [16] dropped age weighting and time discounting from the original 1994 definition of DALYs [55,56], which results in a higher relative weight of young children compared to adults. Table 8 shows a summary of DALYs estimated for Mexico using the original definition of DALYs (WHO method) [55] for comparison with past estimates, and the new GBD 2010 method [16]. The latter method results in less conservative estimates of disease burden, and higher relative weights of fatal cases (YLL) in the total DALY estimates (YLL represented about 57% on average of total DALYs; 50% in 2010 and about 68% in 2011). Most of the years lost to disability (85%), YLD, were due to ambulatory episodes of dengue. The numbers in parentheses indicate the region of uncertainty around base-case estimates (95% certainty levels). Uncertainty in DALYs is driven by the probabilistic distribution of EFs and the duration of hospitalized and ambulatory dengue episodes (Table 5).

**Table 8:** Dengue disease burden estimates in Mexico per million population.

	2010	2011	Average
*WHO method*			
YLL	22.3	20.8	21.5
YLD—ambulatory	54.3	19.5	36.9
YLD—hospitalized	6.9	6.4	6.7
DALYs	83.5	46.7	65.1
(95%CL)	(41.8–131.9)	(29.4–66.3)	(36.0–98.7)
*GBD 2010 method*			
YLL	49.1	45.7	47.4
YLD—ambulatory	44.3	16.1	30.2
YLD—hospitalized	5.7	5.2	5.4
DALYs	99.0	67.0	83.0
(95%CL)	(60.2–142.9)	(45.4–91.7)	(53.2–116.6)

Notes: YLL denotes Years of Life Lost, YLD denotes Years Lost due to Disability, and DALYs denote Disability-Adjusted Life-Years. The *WHO method* refers to the original definition of DALYs proposed by Murray et al. in 1994 [55,56], and used subsequently for most burden of disease estimates. The parameters used (age weights, disability weight, and discount rate) were based on previous studies, for comparability [44,52,91]. The *GBD 2010 method* refers to an updated definition of DALYs used in the Global Burden of Disease (GBD 2010 study) [16], where age weighting and time discounting were dropped from the disease burden estimates. 95% CL denotes a 95% certainty level for each estimate, obtained through Monte Carlo simulations using the probability distributions shown in Table 5. These numbers in parentheses indicate the region of uncertainty around base-case estimates. WHO denotes World Health Organization, YLL denotes years of life lost, YLD denotes years lived with disability, DALY denotes disability adjusted life-years, CL denotes certainty level.

**Fig 4:**
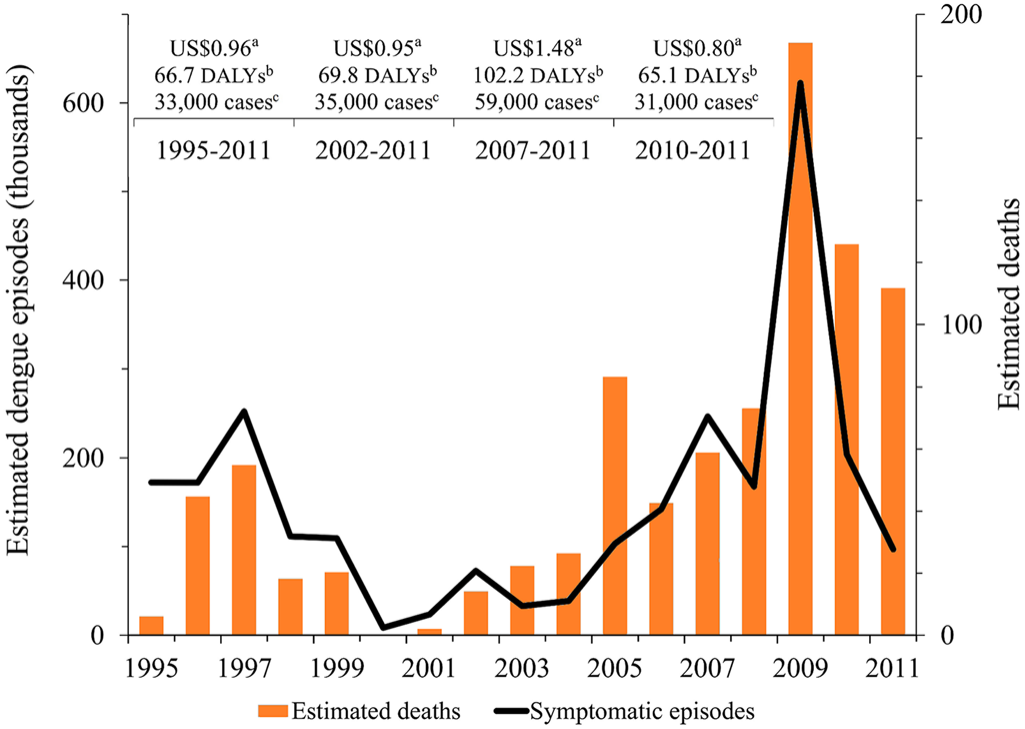
Estimated symptomatic and fatal dengue episodes and economic and disease burden, 1995–2011. Notes: Estimated numbers of dengue episodes are based on refined surveillance data using expansion factors from the Morelos prospective cohort study.^a^ Cost estimates correspond to the average annual economic burden per capita for dengue illness and death (in 2012 US dollars) and do not include surveillance and vector control costs.^b^ Disability adjusted life years (DALYs) are per million population.^c^ Average number of cases of symptomatic dengue infection reported to the Ministry of Health (not adjusted for underreporting).

**Extrapolation of dengue burden using historical data.** If we assume that the age distribution of dengue episodes, proportion of ambulatory and hospitalized patients, overall fatality rates, and the reporting ratios of ambulatory and hospitalized cases in 2010–2011 are on average representative of the situation of dengue in Mexico in the previous years, we can estimate approximate economic and disease burden for those years. While these assumptions might be strong, the objective of this exercise is not to give precise estimates of dengue burden in previous years, but to assess how comparable are 2010–2011 data to historical data. Fig. 4 shows the total estimated number of dengue episodes and economic and disease burden for the previous 5 (2007–2011), 10 (2002–2011), and 17 (1995–2011) years. While the 5-year estimates (2007–2011) were heavily affected by the 2009 outbreak, the average of the annual burden of dengue in 2010–2011 seems a reasonable estimate of the 10-year and 17-year averages.

## Discussion

Dengue imposes a substantial economic and disease burden in Mexico. Because of limited data, combining multiple data sources is a key factor in achieving reliable estimates of dengue burden. Our 2010–2011 average economic and disease burden estimates ($0.80 per capita excluding costs of surveillance and vector control, and 65 DALYs per million population) are below the previous 95% confidence intervals of US$1.5–4.3 per capita and 82–147 DALYs per million population found for Central America and Mexico [44]. Reasons for our lower estimates include the use of refined, Mexico-specific reporting ratios based on the prospective cohort from Morelos, and dengue’s clustering in coastal and tropical areas [5]. Our estimates for the burden of dengue in 2010–2011 were similar to those obtained for the previous 10 and 17 years, but conservative compared to the average burden of disease in the past 5 years, driven partly by the 2009 outbreak (Fig. 4). In DALYs per million population, during this outbreak year dengue imposed a greater disease burden in Mexico (203) than pre-vaccination rotavirus diarrhea (174) [100].

Other studies have found comparable estimates of the economic and disease burden of dengue in the region. Suaya et al. [46] estimated economic burden of dengue per capita for Brazil ($0.85), Venezuela ($0.71), El Salvador ($0.30), Guatemala ($0.10), and Panama ($0.31). These numbers are underestimated since they were not adjusted for the underreporting of dengue episodes, and did not include vector control and surveillance costs. Halasa et al. [47] estimated an economic burden in Puerto Rico of $3.01 per capita without adjusting for EF, $10.84 using a refined estimate of dengue episodes, and $13.00 per capita including prevention activities, vector control, and surveillance costs. Explanations of the higher burden for Puerto Rico compared to Mexico include the island’s higher GDP per capita ($27,678) compared to Mexico ($9,747), the island-wide distribution of dengue in Puerto Rico, and higher EFs for Puerto Rico (EF_H_: 2.4; EF_A_:10). Armien et al. [45] estimated a per capita economic burden of $6.49, including surveillance and vector control costs, during the 2005 outbreak in Panama (EF_T_:6.0). If we assumed that the characteristics of dengue (e.g. distribution, share of hospitalized cases) during the 2009 dengue outbreak were similar to 2010–2011, our per capita cost estimate of dengue in Mexico would have been $3.99 (95%CL: $3.14-$8.72). Our estimates of the economic burden of dengue per capita in Mexico are within the range of comprehensive cost estimates for Nicaragua [49] ($0.97-$2.49; up to $5.44 in an epidemic year). The study in Nicaragua estimated that disease burden ranged from 99–805 DALYs per million population, which is comparable to our estimates for Mexico. Other estimates of disease burden in the region include Puerto Rico [52] (annual average 1984–1994: 658 DALYs per million population; range 145–1,519), and Brazil [91] (annual average 1986–2006: 22 DALYs per million population; range 14–30).

We found no notification of dengue episodes from the private sector in the cohort study in Morelos, a finding that has been confirmed by local public health officers [27]. The paucity of data from the private sector has been found elsewhere [97], and is possibly among the most critical gaps in estimating the true number of symptomatic DENV infections. One interesting finding in this research relates to the population’s health-seeking behavior. The cohort study in Morelos showed that one third of the participants had not visited a private or public healthcare facility, despite having a symptomatic DENV infection. The questionnaire for dengue patients in 4 hospitals suggests that about 11% of patients had visited a pharmacy seeking treatment at the onset of their dengue episode. These results suggest that milder symptoms of dengue go underreported, which is consistent with previous findings [27,28]. Our refinement of reported dengue episodes using EFs include unreported cases from the private sector, as well as patients with symptomatic episodes who did not seek healthcare. Overall, we found an EF for all symptomatic episodes of 4.7 or a reporting ratio of 0.21.

To check the representativeness of our estimated reporting ratio, we compared it with findings from elsewhere in Mexico and neighboring countries. A study in two cities in the state of Tamaulipas [101], near the Texas-Mexico border, suggests that the number of DENV infections represent about 20 times the number of notified cases between 1980 and 2007. Considering that 39% of the DENV infections in the Morelos cohort were symptomatic, using these numbers from Tamaulipas, we would obtain an overall reporting ratio for symptomatic cases of 0.13 (EF_T_ = 7.8). Although the quality of the health system for Morelos is not too different from that for Mexico overall [65], a dengue awareness and education campaign in Morelos may have increased its reporting ratios compared to the rest of the country [33]. Findings from other countries in the Americas [44] found a reporting ratio of 0.08 (EF_T_ = 11.9) for total, 0.43 for hospitalized (EF_H_ = 2.3), and 0.07 for ambulatory (EF_A_ = 15) cases. As the Mexican data are recent and reflect the EPS, which facilitated reporting and increased the quality of data [8,9], the higher reporting ratios are also considered reasonable. Reporting ratios vary in time and by region [26]; hence, our estimate was based on the two years of the cohort study to provide a more stable estimate. Had we considered only post-enrollment infections, we would have obtained a reporting ratio of 0.13 (EF = 8.0, derived from Table 1) and a point estimate of the economic burden of dengue of $253 million or $2.32 per capita (which is included within our 95% certainty level). We think our current estimate of $170 million ($1.56 per capita) is statistically more stable and accurate, although our expansion factor may be an underestimate in relation with other parts of the country with less dengue awareness, or lower overall quality of the health system.

Our estimates suggest that at least 48.9% of the economic burden of dengue corresponds to surveillance and vector control. This share of total costs is higher than those of previous estimates of vector control in other countries. For example, the surveillance and vector control shares of estimated annual economic burden of dengue were 17% in Puerto Rico [47], 30% in Panama [45], and 28% in Thailand [54]. However, the per capita costs of surveillance and vector control (in 2012 US dollars) were lower in Mexico ($0.76) than these other countries ($2.14 in Puerto Rico, $1.79 in Panama, and $1.15 in Thailand). This pattern is partly explained by Mexico’s lower share of the national population at risk of dengue, as the disease is clustered mainly in Mexico’s coastal and tropical regions [5]. Also, Mexico did not experience an outbreak during our study years. Reflecting these patterns, the number of dengue episodes per 1,000 population was lower in Mexico (1.29) than in the other three countries—2.87 in Puerto Rico [47], 9.76 in Panama [45], and 4.08 in Thailand [54].

Several areas of uncertainty in our estimates of disease and economic burden of dengue in Mexico deserve attention. First, estimating the total episodes dengue is difficult due to paucity of data. For example, the cohort study in Morelos showed that 26% of the participants sought care in the private sector; but this estimate may be low as data from the Mexican National Health and Nutrition Survey 2012 showed that about 39% of all outpatient visits (for any illness) were in the private sector [102]. Second, the Morelos cohort is limited in geographical range, calendar years, and age groups, and therefore not necessarily representative of all regions with dengue transmission in Mexico. Local variations in the quality of the health system [34] and accessibility to health services [16] may result in differences in dengue patients’ health-seeking behavior, thus affecting reporting rates of apparent DENV infections. Third, our direct medical costs for dengue episodes were based on macro-costing in two tertiary hospitals in Tabasco, which may not necessarily be representative of hospitals in Mexico. We partially addressed this by adjusting our estimates based on WHO-CHOICE data, and varying our estimates in the sensitivity analysis. Costs in the private sector are probably higher than the costs we used, which possibly makes our economic burden estimates conservative.

Fourth, we only considered surveillance and vector control costs from the federal level. Due to data limitations, we could not distinguish operating and capital expenditures, and did not include allocated and donated resources such as the time allocated by field personnel or volunteers to surveillance and vector control activities, as has been done elsewhere [103]. These limitations make our estimates of costs of surveillance and vector control conservative.

Fifth, despite having improved previous estimates of economic burden by including costs of illness and dengue prevention and control strategies, we did not include other impacts of dengue illness due to data limitations.

Last, our estimates of the burden of dengue were based on the acute and convalescent phases of a dengue episode (Table 5). Recent studies suggest that dengue patients may present long-term symptoms [104–109] like fatigue syndrome or depression, a possibility acknowledged by the WHO since 1997 [110]; unfortunately, there is not enough evidence or agreement on the characteristics (e.g., frequency, intensity, duration) of these persistent symptoms, and whether or not they are caused by dengue alone.

## Conclusions

Dengue costs the Mexican economy an annual average of US$170 (95%CL: 151–292) million, or $1.56 (95%CL: 1.38–2.68) per capita. Of this, $87 (95%CL: 87–209) million or $0.80 per capita (95%CL: 0.62–1.12) corresponds to illness and $83 million or $0.76 per capita to vector control and surveillance. These estimates do not include other costs, such as long-term sequelae of dengue, comorbidities, impacts on travel and tourism, or the disruption of health services during epidemics. Mexico’s annual disease burden from dengue is 65.1 DALYs per million population.

Having objective and comparable estimates of the economic and disease burden of dengue is essential to inform health policy, increase disease awareness, and assess the impact of dengue control technologies [12,111]. More so, considering that several vaccine candidates [112] and other prevention and control technologies [37,40,42,113] are currently under development, and that Mexico might be an early adopter [12,53,114]. Results from the phase III clinical efficacy multicenter trial of a dengue vaccine candidate in the Americas suggest an overall vaccine efficacy of 60.8%, and a reduction in the risk of hospitalization of 80.3% [115]. These recent results make burden estimates even more urgent as Mexico confronts real choices. Effective dengue prevention and control strategies will probably require a combination of approaches and the involvement of various stakeholders [116]. With this study, Mexico joins Panama [45], Puerto Rico [47], Nicaragua [49], and Thailand [54] as the only countries or areas worldwide with comprehensive (illness and preventive) empirical estimates of the cost of dengue. The results from this study reaffirm that exploring approaches to control dengue further would be economically valuable.

## Supporting Information

S1 Table Expenditures of symptomatic patients who did not seek healthcare in a health center or hospital. (PDF)

S1 Checklist STROBE checklist. (PDF)

S1 Text Adult questionnaire. (PDF)
