# Supplementary material for: Economic and Disease Burden of Dengue in Mexico
Source: PLoS Negl Trop Dis. 2015 Mar 18;9(3):e0003547. doi: 10.1371/journal.pntd.0003547 (PMC4364886; doi:10.1371/journal.pntd.0003547)
Supplement: S1 Table — (PDF) [file pntd.0003547.s001.pdf]

Economic and Disease Burden of Dengue in Mexico

Undurraga et al., 2015, *PLoS Neglected Tropical Diseases.*

S1 Table. Expenditures of symptomatic patients who did not seek healthcare in a health center or hospital.

Item	Min (Mex$)	Max (Mex$)	Best (Mex$)	Best (US$)
Patients who sought care in a pharmacy (37% of patients)				
Consultation at pharmacy	0	100	50	3.90
Drugs	30	240	135	10.52
Complete Blood Count (CBC)	100	150	125	9.74
Transport	0	120	60	4.67
Subtotal			370	28.82
Patients who did not seek care in a pharmacy (63% of patients)				
Drugs & remedies at home	30	240	135	10.52
**Total (all patients)**			**222**	**17.23**

Note: Mex$ denotes Mexican pesos. Exchange rate in 2012 was Mex$12.88 equals US$1.00.

The share of patients that did not seek healthcare (30%) was obtained from the Morelos cohort study. We derived the specific costs of homecare by combining data sources. The share of patients who visited a pharmacy (~11%) at the onset of their febrile episode was based on interviews to patients at 4 hospitals in Mexico. The average expenditures from a dengue-related pharmacy visit were based on drug, transport, and diagnostic tests related with an ambulatory visit to a pharmacy, also based on data from interviews to hospitalized patients (S1 Table). Patients were asked about their health-seeking behavior visit before hospitalization, including ambulatory visits to various types of healthcare providers, including pharmacies, traditional healers, hospitals, health centers, laboratories, etc. (S1 Text). We assumed that the patients who stayed at home had non-medical costs comparable to the costs of medications of those who visited a pharmacy (~US$10.52). Uncertainty in these estimates was addressed through a probabilistic sensitivity analysis.
