# Supplementary material for: Economic and Disease Burden of Dengue in Mexico
Source: PLoS Negl Trop Dis. 2015 Mar 18;9(3):e0003547. doi: 10.1371/journal.pntd.0003547 (PMC4364886; doi:10.1371/journal.pntd.0003547)
Supplement: S1 Text — (PDF) [file pntd.0003547.s003.pdf]

Economic and Disease Burden of Dengue in Mexico

Undurraga et al. 2015, *PLoS Neglected Tropical Diseases*.

S1 Text. Adult questionnaire.

The data obtained from this questionnaire were given to Brandeis under a Data Use Agreement between the Carlos Slim Health Institute and the Schneider Institutes for Health Policy, Brandeis University. The Carlos Slim Health Institute provided Brandeis University with a person-level limited dataset containing selected variables from the interviews and abstraction of medical records from four major hospitals in Mexico. The dataset contained no names, addresses, phone numbers, or national identification numbers of any patients of family members. No Protected Health Information (PHI) came to Brandeis University, and none of the data related with medical treatment within the United States of America. Brandeis University established appropriate administrative, technical and physical safeguards to protect the confidentiality of the data and to prevent unauthorized use and/or access to the data.

Two questionnaires were used for the study: an adult and child questionnaire. The only difference was that the child questionnaire was completed by a patient proxy, usually a parent or another caregiver, so some of the questions were phrased slightly differently. The original questionnaires also included a section on quality of life associated with the dengue episode based on a survey designed by the EuroQol Group Foundation (EQ-5D-3L, validated Spanish version for Mexico, www.euroqol.org). The use of this survey was authorized by the EuroQol Group Foundation, but Brandeis is not allowed to include it in the supplementary material for this article due to copyright restrictions. However, the results from this quality of life section were not used to derive economic estimates of the burden of dengue reported in this article.

The research methods and cost questionnaires used in this study were approved by the Brandeis University Committee for Protection of Human Subjects.

Cuestionario Dengue: Pacientes Adultos

Identificación Paciente (ID) ______________
Fecha ______________
Nombre entrevistador ______________

Entrevistador

Q0. ¿Cómo está administrando esta entrevista? (Elija uno)

0 En persona, en centro/ lab de salud
1 En persona, en otro lugar
2 Por teléfono

Q00. El nombre del lugar en el cual el paciente fue reclutado es (elija uno):

1 Hospital Gral. de Mérida, Agustín O'Horán
2 Hospital General de Cancún
3 Hospital General de Cuernavaca
4 Hospital General de Monterrey

Q000. *Entrevistado*

1 Entrevista con el (la) propio(a) paciente
2 Apoderado paciente (use cuestionario menores)

Demografía

Q1. ¿Cuántos años cumplió en su último cumpleaños? _____

999 No sabe
998 No contesta

Q2. *Por favor anote el sexo del paciente*

1 Masculino
2 Femenino

Q3. ¿Cuál es su fecha de nacimiento (día/mes/año)?

__/__/____ dd/mm/aaaa
01/01/2097 No sabe
01/01/2098 No contesta

Nota: *Calcule la edad del paciente. Asegúrese de que la edad calculada es la misma que la reportada por el paciente.*

Calidad de Vida

***Lea lo siguiente**:* Ahora hablaremos del período en que ha estado enfermo con dengue. Le voy a mostrar un calendario para ayudarle a contestar las siguientes preguntas.
Muestre el calendario al paciente, y utilícelo hasta la pregunta Q37. Marque claramente la fecha de hoy en el calendario.

Q4. ¿Cuándo comenzó a sentirse enfermo(a)?
Anote "comienzo dengue" en la fecha indicada en el calendario

__/__/____ dd/mm/aaaa
01/01/2097 No sabe
01/01/2098 No contesta

Q5. ¿Ha tenido fiebre algún día desde [Respuesta Q. 4] hasta hoy?

1 Si
0 No ***Pase a Q9***
8 No contesta ***Pase a Q9***

Q.6 ¿Cuándo comenzó su fiebre? __/__/____ dd/mm/aaaa
Anote "comienzo fiebre" en esa fecha en el calendario 01/01/2097 No sabe
01/01/2098 No contesta

Q.7 ¿Terminó su fiebre?
1 Si
0 No **Pase a Q9**
8 No contesta **Pase a Q9**

Q.8 ¿Cuándo terminó su fiebre? __/__/____ dd/mm/aaaa
Anote "fiebre terminó" en esa fecha en el calendario 01/01/2097 No sabe
01/01/2098 No contesta

Q9. ¿Qué otros síntomas tuvo?
1 Mialgia
2 Nausea
3 Dolor de cabeza
4 Otros

Q9a Otros síntomas____
____
____

Q10. Durante toda la enfermedad, ¿en qué día se sintió peor?
Anote "peor día" en esa fecha en el calendario __/__/____ dd/mm/yyyy
01/01/2097 No sabe
01/01/2098 No contesta

Q11. En este momento, ¿se sigue sintiendo enfermo(a)?
1 Si **Pase a Q14**
0 No

Q12. ¿Se recuperó completamente de este período de enfermedad?
1 Si
0 No **Pase a Q14**
8 No contesta **Pase a Q14**

Q13. ¿Cuándo se recuperó de este período de enfermedad?
Anote "recuperado completamente" en esa fecha en el calendario __/__/____ dd/mm/aaaa
01/01/2097 No sabe
01/01/2098 No contesta

Q14. Después de que comenzó a sentirse enfermo(a), ¿cuán pronto recibió cuidados médicos? (Elija uno)
1 Antes de 24 horas
2 Entre 24 y 48 horas
3 Después de 48 horas
7 No sabe
8 No contesta

Por favor lea: Ahora me gustaría hacerle algunas preguntas sobre su calidad de vida desde [Respuesta a Q4] hasta hoy.
Muestre la forma 2

Q15. ¿Cómo evaluaría usted su salud antes del día en que se comenzó a sentir enfermo(a) en [Respuesta a Q4]? (elija una)

1 Excelente
2 Muy bien
3 Bien
4 Mal
5 Muy mal
8 No contesta

Por favor lea: Para el próximo grupo de preguntas, me gustaría que se concentrara solamente en los días que estuvo enfermo(a).

Si el paciente no se ha recuperado completamente, el entrevistador debe considerar desde que el paciente comenzó a sentirse enfermo(a) hasta el día de la entrevista (hoy).
Si el paciente ya se recuperó completamente, el entrevistador debe considerar desde que el paciente comenzó a sentirse enfermo(a) hasta el día de su recuperación.

Para cada una de las preguntas siguientes, por favor recuerde al paciente los días que estuvo enfermo(a), mostrándole los días marcados de enfermedad en el calendario.

Por favor lea: Ahora vamos a discutir sobre los días que estuvo enfermo.

Muestre las formas 1 y 2.

16. ¿Cómo evaluaría usted su salud durante los días que estuvo enfermo(a)? (elija uno)
Muestre al paciente los días que estuvo enfermo en el calendario.

1 Excelente
2 Muy buena
3 Buena
4 Mala
5 Muy mala
8 No contesta

Q17. Del total de días en que estuvo enfermo(a), ¿durante cuántos días se sintió ‘mal’ o ‘muy mal’? _______

97 No sabe
98 No contesta

Por favor muestre al paciente la forma 3, y recuérdele que determine el número de días que estuvo enfermo(a). El paciente debe completar esta forma.

[The Quality of Life section based on EuroQol Group Foundation EQ-5D-3L, validated Spanish version for Mexico was removed from this version of the survey due to copyright restrictions]

Cuidados recibidos por el paciente desde que comenzó a sentirse mal hasta hoy

Muestra el calendario al paciente (forma 1)
Por favor lea: En el siguiente grupo de preguntas me gustaría saber si usted a pasado uno o más días en un hospital (o centro de salud).

Hospitalización (servicios internos)

Q38. ¿Fue usted hospitalizado mientras duró su enfermedad?

1 SI
0 No ***Pase a las instrucciones antes de Q53***
8 No contesta ***Pase a las instrucciones antes de Q53***

Q39. ¿Puede indicarme el nombre del hospital en cual pasó la mayor cantidad de días hospitalizado?
____

Por favor lea: Ahora me gustaría hacerle algunas preguntas sobre la duración de su hospitalización, los pagos de tratamiento que tuvo que efectuar de su bolsillo, y otros costos relacionados a la hospitalización.

Q40. Durante la hospitalización, ¿cuántas noches pasó en el hospital?

__.__
998 No contesta

Q41. Durante su hospitalización, ¿pasó alguna noche en la unidad de tratamiento intensivo (UTI)?

1 Si
0 No ***Pase a las instrucciones antes de Q43***
8 No contesta ***Pase a las instrucciones antes de Q43***

Q42. ¿Cuántas noches pasó en la unidad de tratamiento intensivo? (*Si el paciente estuvo en la unidad de tratamiento intensivo por una noche o menos, anote "1")*

__.__
998 No contesta

Por favor lea: Una institución "pública" es una institución operada por cualquier nivel de gobierno, y una institución "no publica" es una institución que no es operada de ninguna forma por el gobierno.

Q43. El hospital en el que usted estuvo internado(a), ¿es público o no-público?(elija una)

1 Público
2 No-público
7 No sabe
8 No contesta

Q44. Indique el tipo de hospital en el cual usted pasó la mayor cantidad de noches. (elija uno)

1 Universitario, terciario, o de especialidad
2 Otro hospital
7 No sabe
8 No contesta

Por favor lea: Ahora le voy a preguntar por pagos que tuvo que hacer de su bolsillo.

Los pagos de su bolsillo son el total de pagos que realizó usted o su familia durante el período en el que estuvo enfermo para su tratamiento, independiente de si recibió o no un reembolso del total o parte de este monto.

Q45. Por favor indíqueme su mejor estimación de los gastos de su bolsillo que ha hecho hasta hoy por servicios entregados durante su hospitalización. Por favor incluya tanto los pagos que ya ha efectuado como los que espera por los servicios que ha recibido. (*Si no se han efectuado pagos, ingrese 0.00)*

____

0000000	Cero	***Pase a las instrucciones antes de Q50***
9999997	No sabe	***Pase a las instrucciones antes de Q50***
9999998	No responde	***Pase a las instrucciones antes de Q50***

Q46. ¿Puede usted diferenciar estos pagos según el tipo de servicios que recibió durante su estadía en el hospital?

1	Si	
0	No	***Pase a las instrucciones antes de Q50***
7	No sabe	***Pase a las instrucciones antes de Q50***
8	No responde	***Pase a las instrucciones antes de Q50***
9	No se aplica	

Q47. Pagos de su bolsillo por la suma de costos de consulta y de alta médica
Si no hubo gastos, anote "0.00"

____

99999997	No sabe
99999998	No responde
99999999	No se aplica

Q48. Pagos de su bolsillo por pruebas de diagnóstico, tales como exámenes de sangre, test de dengue, rayos-x, o ecografía
Si no hubo gastos, anote "0.00"

____

99999997	No sabe
99999998	No responde
99999999	No se aplica

Q49. Pagos de su bolsillo por el total de tratamientos como medicamentos, fluidos intravenosos, equipos médicos, o banco de sangre.
Si no hubo gastos, anote "0.00"

____

99999997	No sabe
99999998	No responde
99999999	No se aplica

Por favor lea: Ahora me gustaría hacerle otras preguntas acerca de otros gastos relacionados con el período desde el día en que comenzó a sentirse enfermo [Respuesta a Q4] hasta hoy, como gastos en transporte, comida, o alojamiento. Por favor también incluya todos los gastos efectuados por otros miembros de su hogar (las personas con las que usted vive) que lo han acompañado en el hospital para cuidarlo(a) o ayudarlo(a).

Q50. Por favor indique su mejor estimación de gastos de su bolsillo en transporte hacia y desde el hospital, incluyendo el transporte en ambulancia si se aplica.
Si no hubo gastos, anote"0.00"

Si el paciente o su familia tienen algún vehículo, estime los km viajados (ida y vuelta). Al efectuar las estimaciones de costo se imputará un valor promedio por km recorrido en automóvil, motocicleta, o bicicleta.

________________	
99999997	No sabe
99999998	No responde
99999999	No se aplica

Q51. Por favor indique su mejor estimación de gastos de su bolsillo en comida.
Si no hubo gastos, anote “0.00”

________________	
99999997	No sabe
99999998	No responde
99999999	No se aplica

Q52. Por favor indique su mejor estimación de gastos de su bolsillo en alojamiento en alguna institución que no sea de salud, como un hotel.
Si no hubo gastos, anote “0.00”

________________	
99999997	No sabe
99999998	No responde
99999999	No se aplica

Cuidados Ambulatorios

Por favor lea: Las siguientes preguntas son sobre los cuidados ambulatorios que usted recibió durante su período de enfermedad, desde que comenzó a sentirse mal [Respuesta a Q4] hasta hoy.
Incluya cuidados de todos los tipos de instituciones de salud, incluyendo farmacias, consultas privadas, curanderos tradicionales, centros de salud, postas, consultas ambulatorias en hospitales, salas de emergencia, y/o laboratorios.

Q53. ¿Visitó usted alguna institución de salud durante el período de enfermedad?

1	Si	
0	No	***Pase a las instrucciones antes de Q66***
8	No contesta	***Pase a las instrucciones antes de Q66***

Servicios ambulatorios recibidos durante el período de enfermedad, desde el día que comenzó a sentirse enfermo(a) hasta hoy

Por favor lea: Piense en cada vez que ha contactado alguna de las siguientes instituciones o proveedores de salud, durante el período en que estuvo enfermo. Luego especifique el tipo de atención que recibió en cada institución or proveedor que visitó

(consulta, pruebas de diagnóstico, y/o tratamiento) e intente recordar los montos que pagó de su bolsillo en total o para cada tipo de servicio recibido.

Q54. Escoja de la siguiente lista la primera (*segunda, tercera, …)* tipo de institución de salud que usted contactó durante el período en el que estuvo enfermo (muestra la forma 4). Escoja una institución para cada visita.

Visita	Fecha	Tipo de institución o proveedor								
		Farmacia (01)	Curandero tradicional (02)	Consulta doctor (03)	Centro de salud (04)	Cuidados ambulatorios en hospital (05)	Sala de emergencia (06)	Laboratorio (07)	No sabe (97)	No responde (98)
Primera										
Segunda										
Tercera										
Cuarta										
Quinta										
Sexta										

Por favor lea: Una institución "pública" es una institución operada por cualquier nivel de gobierno y una institución "no-pública" es cualquier institución que no es operada por el gobierno.

Q55. ¿Qué tipo de institución visitó durante su *XX* visita? ¿Era una institución pública o no-pública? (elija una por visita)

Visita	Pública (1)	Non-pública (2)	No sabe (7)	No responde (8)
Primera				
Segunda				
Tercera				
Cuarta				
Quinta				
Sexta				

Q56. ¿Qué tipo de servicios recibió usted en esta institución durante su *XX* visita? (marque todos lo que apliquen)

Visita	Consulta (01)	Pruebas de diagnóstico, como rayos x, examen de sangre (02)	Tratamientos como medicaciones, fluidos intravenosos (03)	No sabe (07)	No responde (08)
Primera					
Segunda					
Tercera					
Cuarta					
Quinta					
Sexta					

Por favor lea: Ahora me gustaría preguntarle sobre los gastos de su bolsillo que usted efectuó en esta institución.
Cuando sea posible, chequee el monto con las boletas o recibos disponibles de la institución de salud.

Q57. Por favor reporte su mejor estimación del total de gastos de su bolsillo que efectuó por el tratamiento recibido en esta institución. Por favor incluya los pagos que ya ha realizado, y también los que espera realizar por los servicios recibidos. *Si no hubo gastos, anote "0.00"*

Visita	Monto $ xxx.xx	***Si el monto es 0, pase a las instrucciones antes de Q62***	No sabe (9999997)	***Pase a las instrucciones antes de Q62***	No contesta (9999998)	***Pase a las instrucciones antes de Q62***
Primera						
Segunda						
Tercera						
Cuarta						
Quinta						
Sexta						

Q58. ¿Podría identificar los pagos totales que realizó por tipo de servicios recibidos?

Visita	Si (1)	No (0)	***Pase a las instrucciones antes de Q62***	No contesta (8)	***Pase a las instrucciones antes de Q62***	No se aplica (9)
Primera						
Segunda						
Tercera						
Cuarta						
Quinta						
Sexta						

Q59. Pagos de su bolsillo por: consulta
Si no hubo gastos, anote "0.00"

Visita	Monto, $ xxx.xx	No sabe (9999997)	No responde (9999998)	No se aplica (9999999)
Primera				
Segunda				
Tercera				
Cuarta				
Quinta				
Sexta				

Q60. Pagos de su bolsillo por: pruebas de diagnóstico o banco de sangre, como pruebas de dengue, rayos-x, ecografías, exámenes de sangre,…
Si no hubo gastos, anote "0.00"

Visita	Monto, $ xxx.xx	No sabe (9999997)	No contesta (9999998)	No se aplica (9999999)
Primera				
Segunda				
Tercera				
Cuarta				
Quinta				
Sexta				

Q61. Pagos de su bolsillo por: tratamientos tales como fluidos intravenosos, medicaciones y remedios, equipos médicos.
Si no hubo gastos, anote "0.00"

Visita	Monto, $ xxx.xx	No sabe (9999997)	No contesta (9999998)	No se aplica (9999999)
Primera				
Segunda				
Tercera				
Cuarta				
Quinta				
Sexta				

Por favor lea: Ahora me gustaría hacerle algunas preguntas sobre otros gastos que usted haya efectuado en esta institución durante el período en el que estuvo enfermo, tales como gastos en transporte, comida, y alojamiento. Por favor incluya los gastos realizados por cualquier otro miembro de su hogar que lo haya acompañado en esta primera (*segunda, tercera)* institución de salud.

Q62. Por favor indique su mejor estimación de gastos de su bolsillo en transporte (hacia y desde la institución de salud), incluyendo la ambulancia si se aplica.
Si el paciente o su familia tienen algún vehículo, estime los km viajados (ida y vuelta). Al efectuar las estimaciones de costo se imputará un valor promedio por km recorrido en automóvil, motocicleta, o bicicleta. Si no hubo gastos, anote "0.00".

Visita	Monto, $ xxx.xx	No sabe (9999997)	No responde (9999998)	No se aplica (9999999)
Primera				
Segunda				
Tercera				
Cuarta				
Quinta				
Sexta				

Q63. Por favor indique su mejor estimación de gastos de su bolsillo en comida
Si no hubo gastos, anote "0.00"

Visita	Monto, $ xxx.xx	No sabe (9999997)	No contesta (9999998)	No se aplica (9999999)
Primera				
Segunda				
Tercera				
Cuarta				
Quinta				
Sexta				

Q64. Por favor indique su mejor estimación de gastos de su bolsillo en alojamiento en una institución no relacionada a la salud, como un hotel.
Si no hubo gastos, anote "0.00"

Visita	Monto, $ xxx.xx	No sabe (9999997)	No contesta (9999998)	No se aplica (9999999)
Primera				
Segunda				
Tercera				
Cuarta				
Quinta				
Sexta				

Q65. ¿Visitó usted alguna otra institución de salud durante el período desde que comenzó a sentirse enfermo(a) [Respuesta a Q4] hasta ahoy?

1	Si	***Repita el proceso comenzando desde la Q54, hasta Q65***
0	No	

Impacto de la enfermedad en el hogar, desde el día que comenzó a sentirse enfermo(a) [*Respuesta a Q4*] hasta hoy

Por favor lea: Me gustaría hacerle algunas preguntas sobre cómo lo(la) afectó el período en que usted estuvo enfermo (a) a usted y los miembros de su hogar. Se consideran miembros de su hogar a todas aquellas personas que viven con usted y con quienes comparte su comida. Un miembro de su hogar se ve afectado si él o ella gastan más dinero del habitual para cuidarle a usted, o pierde horas de trabajo, ingreso, o educación debido a su enfermedad.

Por favor lea: Hablemos sobre usted primero

El paciente

Q66. ¿Cuál es el más alto nivel de educación que usted ha completado? (elija uno)
Muestre la forma 5

01	Analfabeto(a)
02	Educación inicial
03	Educación primaria
04	Educación secundaria, no graduado(a)
05	Educación secundaria completa
06	Educación media superior (preparatoria) no graduado
07	Educación media superior (preparatoria) completa
08	Educación universitaria incompleta
09	Educación universitaria completa
98	No contesta

Q67. ¿Está estudiando actualmente?

1	Si	
0	No	***Pase a Q70***
8	No contesta	***Pase a Q70***

Q68. ¿Ha perdido algún día de educación debido a su enfermedad?

1	Si	
0	No	***Pase a Q70***
8	No contesta	

Q69. ¿Cuántos días de educación ha perdido debido a su enfermedad?
Anote "0" si ninguno

_____

97	No sabe
98	No responde
99	No se aplica

Q70. ¿Tiene ingresos por su trabajo?

1 Si
0 No ***Pase a Q76***
8 No contesta

Q71. ¿Ha perdido algún día de trabajo o ingresos debido a su enfermedad?
Si el (la) paciente declara no haber perdido ningún día de trabajo, por favor confirme que él o ella no dejó de percibir ingresos ese día.

1 Si
0 No ***Pase a Q76***
7 No sabe ***Pase a Q76***
8 No contesta ***Pase a Q76***

Q72. ¿Cuántos días de trabajo perdió debido a su enfermedad?
Anote "0" si ninguno

_____
97 No sabe
98 No contesta
99 No se aplica

Q73. ¿Cuántos ingresos dejó de percibir debido a su enfermedad? (Por favor dé su mejor estimación)

____
99999997 No sabe
99999998 No contesta
99999999 No se aplica

Q74. ¿Recibió algún pago de licencia por enfermedad para compensar los días en los que no trabajó mientras estuvo enfermo(a)? (puede ser pagado por el empleador, seguro de enfermedad, etc).

1 Si
0 No ***Pase a Q76***
8 No contesta ***Pase a Q76***

Q75. ¿Cuántos días de pago de licencia por enfermedad recibió?
Anote "0" si ninguno

_____
97 No sabe
98 No contesta
99 No se aplica

Miembros del hogar

Q76. ¿Cuántas personas viven en su hogar? (además de usted)

_____
00 cero ***Pase a las instrucciones antes de Q94***
98 No contesta

Q77. ¿Cuántos de esos miembros de su hogar se visto afectados(as) directamente por su enfermedad?
Por favor lea: Un miembro de su hogar se ve afectado(a) directamente por su enfermedad si ella o él ha gastado más tiempo o dinero del usual cuidando de usted, o ha perdido horas de trabajo, ingresos, o horas de educación debido a su enfermedad.

______
00 Cero
98 No contesta

Por favor lea: Ahora me gustaría hacerle algunas preguntas sobre los miembros de su hogar que se han visto afectados(as) por su enfermedad.

Cuidadores

Q78. ¿Qué relación tiene usted con esta persona (primera, segunda, etc) que se ha visto afectado(a) por su enfermedad? (elija una)
Muestre forma 6

Relación	Miembro del hogar							
	Primer miembro	Segundo miembro	Tercer miembro	Cuarto miembro	Quinto miembro	Sexto miembro	Séptimo miembro	Octavo miembro
Madre (01)								
Padre (02)								
Esposo (03)								
Esposa (04)								
Pareja (05)								
Hermana (06)								
Hermano (07)								
Hijo (08)								
Hija (09)								
Abuela (10)								
Abuelo (11)								
Otro (12)								
No contesta (98)								

Q79. ¿Qué edad tiene [*miembro del hogar*]?

Miembro del hogar	Edad en años	No sabe (997)	No contesta (998)
Primer miembro			
Segundo miembro			
Tercer miembro			
Cuarto miembro			
Quinto miembro			
Sexto miembro			
Séptimo miembro			
Octavo miembro			

Q80. ¿Cuál fue el nivel de educación más alto que [*miembro del hogar*] completó? (Elija uno) *Muestra forma 5*

Escolaridad	Miembro del hogar							
	Primer miembro	Segundo miembro	Tercer miembro	Cuarto miembro	Quinto miembro	Sexto miembro	Séptimo miembro	Octavo miembro
01 Analfabeto								
02 Inicial								
03 Primaria								
04 Secundaria no graduado								
05 Secundaria completa								
06 Media superior no graduado								
07 Media superior completa								
08 Universitaria incompleta								
09 Universitaria completa								
98 No contesta								

Q81. ¿Está [*miembro del hogar*] estudiando actualmente?

Miembro del hogar	Si (01)	No (02)	***Pase a Q84***	No sabe (07)	***Pase a Q84***	No contesta (08)	***Pase a Q84***
Primer miembro							
Segundo miembro							
Tercer miembro							
Cuarto miembro							
Quinto miembro							
Sexto miembro							
Séptimo miembro							
Octavo miembro							

Q82. ¿Ha perdido [*miembro del hogar*] algún día de educación debido a su enfermedad?

Miembro del hogar	Si (01)	No (02)	***Pase a Q84***	No sabe (07)	***Pase a Q84***	No contesta (08)	***Pase a Q84***
Primer miembro							
Segundo miembro							
Tercer miembro							
Cuarto miembro							
Quinto miembro							
Sexto miembro							
Séptimo miembro							
Octavo miembro							

Q83. ¿Cuántos días de educación perdió [*miembro del hogar*] debido a su enfermedad? *Anote "0" si ninguno*

Miembro del hogar	Días de educación perdidos	No sabe (97)	No contesta (98)
Primer miembro			
Segundo miembro			
Tercer miembro			
Cuarto miembro			
Quinto miembro			
Sexto miembro			
Séptimo miembro			
Octavo miembro			

Q84. ¿Está [*miembro del hogar*] recibiendo ingresos por su trabajo?

Miembro del hogar	Si (01)	No (02)	***Pase a Q90***	No sabe (07)	***Pase a Q90***	No contesta (08)	***Pase a Q90***
Primer miembro							
Segundo miembro							
Tercer miembro							
Cuarto miembro							
Quinto miembro							
Sexto miembro							
Séptimo miembro							
Octavo miembro							

Q85. ¿Ha perdido algún día de trabajo o ingresos [*miembro del hogar*] debido a su enfermedad?
Si el [*miembro del hogar] no perdió ningún día de trabajo, por favor confirme que no perdió ingresos tampoco.*

Miembro del hogar	Si (01)	No (02)	***Pase a Q90***	No sabe (07)	***Pase a Q90***	No contesta (08)	***Pase a Q90***
Primer miembro							
Segundo miembro							
Tercer miembro							
Cuarto miembro							
Quinto miembro							
Sexto miembro							
Séptimo miembro							
Octavo miembro							

Q86. ¿Cuántos días de trabajo perdió [*miembro del hogar*] debido a su enfermedad?*Anote "0" si ninguno*

Miembro del hogar	Número de días	No sabe (Code:97)	***Pase a Q90***	No contesta (98)	***Pase a Q90***	No se aplica (99)	***Pase a Q90***
Primer miembro							
Segundo miembro							
Tercer miembro							
Cuarto miembro							
Quinto miembro							
Sexto miembro							
Séptimo miembro							
Octavo miembro							

Q87. ¿Cuántos ingresos perdió [*miembro del hogar*] debido a su enfermedad? (Por favor indique su mejor estimación)
Si no hubo pérdida de ingresos, anote "0.00".

Miembro del hogar	Monto, $xx.xxx	No sabe(9999997)	No contesta (9999998)	No se aplica (9999999)
Primer miembro				
Segundo miembro				
Tercer miembro				
Cuarto miembro				
Quinto miembro				
Sexto miembro				
Séptimo miembro				
Octavo miembro				

Q88. ¿Recibió [*miembro del hogar*] algún pago de licencia por enfermedad para compensar por los días en que no trabajó mientras estuvo enfermo? (puede ser pagado por el empleador, seguro de enfermedad, etc)

Miembro del hogar	Si (01)	No (02)	***Pase a Q90***	No sabe (07)	***Pase a Q90***	No contesta (08)	***Pase a Q90***	No aplica (09)	***Pase a Q90***
Primer miembro									
Segundo miembro									
Tercer miembro									
Cuarto miembro									
Quinto miembro									
Sexto miembro									
Séptimo miembro									
Octavo miembro									

Q89. ¿Cuántos días de pago de licencia por enfermedad recibió su [*miembro del hogar*]?

Miembro del hogar	Número de días pagados	No sabe (97)	No contesta (98)
Primer miembro			
Segundo miembro			
Tercer miembro			
Cuarto miembro			
Quinto miembro			
Sexto miembro			
Séptimo miembro			
Octavo miembro			

Q90. De todos los días que estuvo usted enfermo(a), ¿cuántos días en total lo cuidó o acompañó su [*miembro del hogar*]? *Si ninguno, anote "0"*

Miembro del hogar	Número de días	***Si "0" pase a Q92***	No sabe (97)	***Pase a Q92***	No contesta (Code:98)	***Pase a Q92***	No se aplica (Code:99)	***Pase a Q92***
Primer miembro								
Segundo miembro								
Tercer miembro								
Cuarto miembro								
Quinto miembro								
Sexto miembro								
Séptimo miembro								
Octavo miembro								

Por favor lea: En las preguntas a continuación, por favor piense en el total de horas de cuidado adicionales al día que recibió usted de su [*miembro del hogar*] mientras estuvo enfermo(a). Las horas adicionales se refieren al número de horas por sobre las que normalmente su [*miembro del hogar*] le dedica. Al estimar el tiempo, por favor incluya el tiempo que pasó en su hogar, en la institución de salud, y en el transporte hacia y desde la institución de salud.

Q91. Durante estos [*respuesta a Q90*] días, ¿cuál fue, en promedio, el total de horas adicionales que [*miembro del hogar*] pasó cuidando o acompañándole a usted?
Si ninguno, anote 0

Miembro del hogar	Número de días	No sabe (97)	No contesta (98)	No se aplica (99)
Primer miembro				
Segundo miembro				
Tercer miembro				
Cuarto miembro				
Quinto miembro				
Sexto miembro				
Séptimo miembro				
Octavo miembro				

Q92. ¿Tuvo su [*miembro del hogar*] que incurrir en gastos adicionales, como transporte, comida, o alojamiento, debido a su enfermedad?

Miembro del hogar	Si (01)	No (02)	***Pase al próximo miembro del hogar***	No sabe (07)	***Pase al próximo miembro del hogar***	No contesta (08)	***Pase al próximo miembro del hogar***	No se aplica (09)	***Pase al próximo miembro del hogar***
Primer miembro									
Segundo miembro									
Tercer miembro									
Cuarto miembro									
Quinto miembro									
Sexto miembro									
Séptimo miembro									
Octavo miembro									

Q93. Para esta pregunta, por favor declare su mejor estimación del total de gastos adicionales en estos [*respuesta a Q90*] días, no incluidos en las preguntas anteriores.
Si no hubo gastos adicionales, anote "0.00".

Miembro del hogar	Monto, $xx.xxx	No sabe(9999997)	No contesta (9999998)	No se aplica (9999999)
Primer miembro				
Segundo miembro				
Tercer miembro				
Cuarto miembro				
Quinto miembro				
Sexto miembro				
Séptimo miembro				
Octavo miembro				

Revise la Q76, si la respuesta es más de 1, entonces repita las preguntas Q77 a Q93 para todos los miembros del hogar enlistados. Si Q76=1, entonces pase al Q94

Fuentes de financiamiento

Por favor lea: Ahora me gustaría hacerle algunas preguntas sobre las fuentes de financiamiento que usted ha utilizado o que piensa utilizar para pagar por los gastos de salud relacionados a su enfermedad desde que se comenzó a sentir enfermo(a) [Respuesta a Q4] hasta hoy.

Q94. ¿Ha gastado parte de sus ingresos actuales o ahorros o de algún miembro de su familia?

1 Si
0 No
8 No contesta

Q95. ¿Ha pedido dinero prestado a miembros de su familia o amigos que no son parte de su hogar?

1 Si
0 No
8 No contesta

Q96. ¿Ha pedido dinero prestado a alguien que no sea miembro de su familia o amigo para financiar los costos de su enfermedad?

1 Si
0 No
8 No contesta

Q97. ¿Ha vendido o transferido algún elemento de su hogar (por ejemplo, tierra, joyas, vehículos) para financiar los costos de su enfermedad?

1 Si
0 No
8 No contesta

Q98. ¿Ha recibido ayuda financiera de alguna otra institución (por ejemplo, la iglesia, el templo, la comunidad, instituciones de caridad, etc)?

1 Si
0 No
8 No contesta

Q99. ¿Ha recibido atención médica gratuita?

1 Si
0 No
8 No contesta

Q100. ¿Tiene usted un seguro de salud?

1 Si
0 No *Agradezca al paciente y termine la entrevista*
7 No sabe
8 No responde

Q101. ¿Su seguro de salud le reembolsó o le va a reembolsar los gastos médicos que ha pagado o va a pagar de su bolsillo, relacionados con esta enfermedad?

1	Si	
0	No	*Agradezca al paciente y termine la entrevista*
7	No sabe	*Agradezca al paciente y termine la entrevista*
8	No contesta	*Agradezca al paciente y termine la entrevista*

Q102. Considerando todos los gastos pagados de su bolsillo desde que comenzó a sentirse enfermo(a) [respuesta Q4] hasta hoy, ¿Podría usted estimar cuánto le ha reembolsado o va a reembolsarle su compañía de seguros?

____

99999997	No sabe
99999998	No contesta

Q103. ¿Su compañía de seguros le ha reembolsado directamente algún costo a su médico o a la institución médica donde se atendió?

1	Si
2	No
7	No sabe
8	No contesta

Por favor lea: ¡Muchas gracias por su participación en esta encuesta! Si tiene cualquier pregunta relacionada con este estudio, por favor contacte a ______________ en el teléfono ___________ o dirección___________________________.
